# Template-Guided Hierarchical Multi-View Registration Framework of Unordered Bridge Terrestrial Laser Scanning Data

**DOI:** 10.3390/s24051394

**Published:** 2024-02-21

**Authors:** Guikai Xiong, Na Cui, Jiepeng Liu, Yan Zeng, Hanxin Chen, Chengliang Huang, Hao Xu

**Affiliations:** 1 Key Laboratory of New Technology for Construction of Cities in Mountain Area (Ministry of Education), Chongqing University, Chongqing 400045, China; guikaixiong@stu.cqu.edu.cn (G.X.); cuina@cqu.edu.cn (N.C.); yanzg@stu.cqu.edu.cn (Y.Z.); 2 School of Civil Engineering, Chongqing University, Chongqing 400045, China; 3 Chongqing Academy of Surveying and Mapping, Chongqing 401121, China; 4 Technology Innovation Center for Spatio-Temporal Information and Equipment of Intelligent City, Ministry of Natural Resources, Chongqing 401121, China

**Keywords:** point cloud registration, hierarchical multi-view registration, terrestrial laser scanning, template-guided, bridge point cloud data

## Abstract

The registration of bridge point cloud data (PCD) is an important preprocessing step for tasks such as bridge modeling, deformation detection, and bridge health monitoring. However, most existing research on bridge PCD registration only focused on pairwise registration, and payed insufficient attention to multi-view registration. In addition, to recover the overlaps of unordered multiple scans and obtain the merging order, extensive pairwise matching and the creation of a fully connected graph of all scans are often required, resulting in low efficiency. To address these issues, this paper proposes a marker-free template-guided method to align multiple unordered bridge PCD to a global coordinate system. Firstly, by aligning each scan to a given registration template, the overlaps between all the scans are recovered. Secondly, a fully connected graph is created based on the overlaps and scanning locations, and then a graph-partition algorithm is utilized to construct the scan-blocks. Then, the coarse-to-fine registration is performed within each scan-block, and the transformation matrix of coarse registration is obtained using an intelligent optimization algorithm. Finally, global block-to-block registration is performed to align all scans to a unified coordinate reference system. We tested our framework on different bridge point cloud datasets, including a suspension bridge and a continuous rigid frame bridge, to evaluate its accuracy. Experimental results demonstrate that our method has high accuracy.

## 1. Introduction

According to the Ministry of Transport of the People’s Republic of China, the total number of highway bridges surpassed one million at the end of 2022 in China [1]. These bridges include large-scale structures that span seas, rivers, and lakes, as well as bridges of various sizes. As the operation time of these bridges increases, issues related to their structural health and carrying capacity have become increasingly prominent [2,3]. Regular operation and maintenance management of bridges are essential to ensure traffic safety, extend the lifespan of bridges, and reduce maintenance costs. There are several methods available to obtain structural dynamic parameters that can reflect the health condition of bridges. One commonly used approach is the eigen perturbation method [4], which enables the extraction of modal parameters. In addition to analytical methods, manual inspections remain essential for certain tasks such as crack inspections, settlement detection, bridge alignment checks. However, traditional manual maintenance methods are expensive, time-consuming, and subjective and relies on inspectors to obtain all information accurately [5].

Recently, the three-dimensional (3D) laser scanning technique has opened up new possibilities for bridge health monitoring and maintenance [6]. The terrestrial laser scanner (TLS) can acquire 3D point cloud data (PCD) of bridges in a short period of time. PCD provides a comprehensive and accurate representation of bridges, allowing for a detailed assessment of its current condition. By analyzing the acquired PCD, valuable insights can be gained to guide maintenance efforts and ensure the longevity and safety of the bridge structure. Furthermore, PCD can be leveraged to create building information modelling (BIM) models. A BIM model provides a digital representation of a bridge and can be valuable for bridge maintenance and digital twins [5,7]. To obtain PCD of the entire bridge, it is necessary to perform multiple scans from different locations because of the limited view of TLS. However, each scan has its own coordinate system, making it essential to align all the scans into a unified coordinate system in order to generate an accurate 3D model of the bridge. Because the accuracy of the 3D model is heavily influenced by the registration accuracy, PCD registration therefore becomes a crucial preprocessing step for TLS-based bridge health monitoring and maintenance.

Although some researchers have studied registration methods of bridge PCD, they mainly focused on pairwise registration [8,9]. However, to align multiple scans of a bridge to a unified coordinate system, the utilization of multi-view registration techniques is essential. In engineering practice, artificial markers such as target spheres [10] and target papers [11] have been commonly used to assist registration and improve registration accuracy when dealing with multiple scans. Although the registration results using these artificial markers are highly reliable, placing artificial markers is time-consuming and costly [12], especially for large infrastructure like bridges. Extensive research has been conducted on marker-free multi-view registration techniques. However, when it comes to applying these techniques to the registration of bridge PCD, there are two notable challenges that need to be addressed. The first challenge involves recovering the overlaps between unordered multiple scans. This typically requires extensive pairwise matching and the creation of a fully connected graph that encompasses all the scans [13]. However, when deal with TLS scans of large bridges, the process of extensive pairwise matching can become quite time-consuming. The second challenge is related to determining the merging order of the scans. Typically, the creation of a fully connected graph that encompasses all the scans is necessary to establish the merging order [14]. However, when dealing with a large number of scans, creating such a fully connected graph can be inefficient. To tackle this challenge, Wu et al. [12] proposed a method to simplify this heavy registration task by subdividing all the scans into several scan-blocks. In this way, the number of pairwise registration procedures is significantly reduced. However, they constructed scan-blocks according to the scanning order, which is sometimes impractical in bridge scenes. Because bridges are essential components of urban transportation infrastructure, minimizing the disruption to urban traffic during the scanning process is crucial. As a result, the PCD of bridge scans cannot always be registered in a sequential order. Manual specification of the registration sequence becomes necessary in such cases. However, for large bridges, where dozens of scans may be acquired, the manual specification process can become quite tedious and inefficient.

To address these issues, this paper proposes a template-guided hierarchical multi-view registration framework that can register unordered bridge terrestrial laser scanning data without any artificial targets. Firstly, the overlaps of multiple scans are recovered using a template-guided initial pose estimation method and extensive pairwise matching is avoided. Next, all the scans are partitioned into different scan-blocks based on their locations and overlaps. Subsequently, the pairwise coarse registration is conducted in each scanning block, and the transformations are obtained using an intelligent optimization algorithm. After that, the fine registration is then performed to further refine the poses within each block. Finally, the above steps are repeated between blocks until all scans are merged into a unified common coordinate system. The main contributions of the proposed method are as follows:

(1) A marker-free multi-view registration framework is proposed to hierarchically align unordered bridge terrestrial laser scanning data.
(2) A template-based initial pose estimation method is proposed to recover the overlaps of unordered PCD, which avoids extensive pairwise matching and improves the efficiency.
(3) To group scans with high overlaps into the same block, a graph partition algorithm based on the overlaps and scanning locations is utilized to construct scan-blocks.

## 2. Research Background

### 2.1. Registration of Bridge PCD

Several researchers have conducted studies on the registration techniques for bridge PCD. For example, Data et al. [8] proposed a method for pairwise registration of bridge PCD. They relied on straight-line edges as linear features and used the random sample consensus (RANSAC) algorithm with a hash table of line pairs to match line pairs. The experiments in three bridge datasets demonstrated its accuracy and efficiency. Zhao et al. [15] proposed an efficient local descriptor for pairwise registration and validated its accuracy using a dataset of a large-scale high-pier concrete bridge. Based on a long-span suspension bridge dataset, Zhang et al. [9] compared and evaluated three iterative closest point (ICP) registration methods from various aspects including convergence rate, execution time, and accuracy. They also provided suggestions for efficiency optimization and accuracy improvement to enhance long-span bridge deformation analysis. Deng et al. [16] introduced a novel dual-purpose target for total station and laser scanner applications. Their experimental results on a long-span arch bridge demonstrated that the using this target can improve the accuracy of PCD registration. While these studies have made significant contributions to the field of bridge PCD registration, it is important to note that they primarily focused on pairwise registration and did not explore multi-view registration methods. However, multi-view registration is more crucial in determining the speed and accuracy of bridge PCD registration.

### 2.2. Multi-View Registration

For unordered multiple scans, multi-view registration first obtains the scanning order of all the scans, and then merges them into a single PCD. The methods for obtaining the scanning order can be roughly classified to marker-based methods and marker-free methods. Marker-based methods involve placing unique markers in the scanning scene and identifying these markers from the scanned PCD to assist with registration. For example, Singh et al. [17] developed 3D unique identifiers along with a 3D registration workflow for mapping and monitoring applications in underground mines. Ge et al. [18] presented an automated marker-based approach for the registration of unordered scans in complex forest scenes, which can automatically detect the artificial markers and build a geometric network to judge their connectivity. However, since installing markers is time-consuming and expensive, these methods are only suitable for complex or single repetitive scenarios. For marker-free methods, Dong et al. [13] utilized multi-level descriptors, including a local descriptor called binary shape context (BSC) and a global descriptor named the vector of locally aggregated descriptor (VLAD), to describe each scan. They evaluated the similarity between every pair of scans based on the distance between their features. Then, they iteratively merged two scans until all the scans were combined into a single coordinate system. However, the performance of VLAD is directly influenced by the distinctiveness of the descriptor. To enhance the robustness of descriptors in urban scenarios dominated by planar structures, Ge et al. [19] proposed a new descriptor by combining panoramic images and the planar features of PCD. However, due to the diverse types of components in bridge scenes, it is challenging to segment them using planar structures, making this method difficult to apply in such scenarios. In addition, the extensive pairwise matching is time-consuming when dealing with a large number of scans.

The methods for merging scans can be roughly divided into sequential registration methods and joint registration methods. Sequential registration methods utilize pairwise registration to iteratively merge two scans until all the scans are merged into a common coordinate system [20]. To determine the merging order, Weber et al. [14] created a fully connected graph and extracted the minimum spanning tree (MST) to define the merging order from root to leaf. The weights of edges in the fully connected graph were defined based on the number of true correspondences between two scans. However, when applied to large-scale scans, searching for corresponding point pairs can be time-consuming. To improve the efficiency of the MST-based methods, some researchers have adopted shape growing algorithms to merge scans, which have higher computational efficiency [19,21]. Other researchers have employed hierarchical merging-based methods to enhance the robustness and efficiency of scans with limited overlaps. For example, Wu et al. [12] proposed a framework for aligning ordered multi-view PCDs. Their approach involves partitioning all the scans into blocks based on the scanning order and then merging the scans within each block to achieve alignment. However, this method is not suitable for unordered scans. Joint registration-based methods consider the multi-view registration problem as a graph optimization problem by using all pairwise registration results to minimize global residuals. Theiler et al. [22] constructed an energy function by considering all loop consistency constraints in the graph and then utilized the lazy flipper algorithm to minimize the energy function, thereby reducing the overall registration error. Tang et al. [23] proposed a method to minimize the sum of squared distances between point pairs generated by the ICP algorithm. Their approach includes a constraint that ensures the product of all transformations within each loop results in an identity matrix. This constraint helps to optimize the alignment process and improve the accuracy of the registration results. However, these methods only redistribute the registration errors on the constructed graph without updating correspondences, and they cannot reduce the total registration error [20].

## 3. Methodology

**Figure 1:**
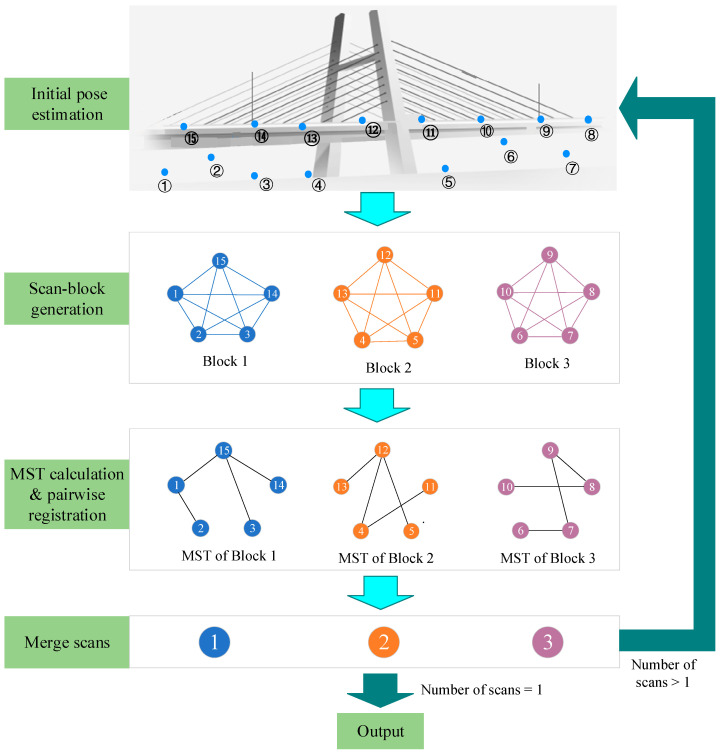
Flowchart of the proposed method.

The proposed method aims to addresses the challenge of automatically aligning unordered scans of bridges without any makers. The overview of the registration framework is plotted in Figure 1. For unordered scans, a template-guided approach is employed in Section 3.1 to recover the overlaps. Subsequently, all the scans are partitioned into different scan-blocks based on their scanning locations and overlaps in Section 3.2. Within each block, pairwise coarse registration is conducted using an intelligent optimization algorithm, as described in Section 3.3. Finally, the fine registration is performed to further refine the poses within each block in Section 3.4. These steps are iteratively executed between blocks until all scans are merged into a unified common coordinate system.

### 3.1. Template-Guided Initial Pose Estimation

Typically, bridges are long and symmetrical structures, and their geometric features on the side view are distinct and can be leveraged to approximate the location of each scan. By comparing the geometric features of the side view of an individual scan of the entire bridge, the approximate position of that scan along the bridge’s length can be determined. Therefore, using the as-designed side view as the template, an initial pose estimation method is proposed.

#### 3.1.1. Acquisition of Side View Geometric Features

**Figure 2:**
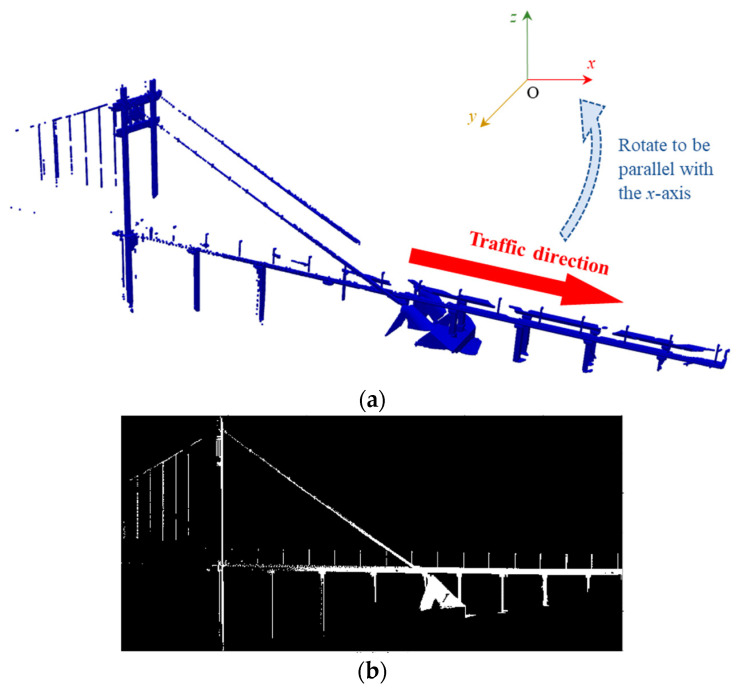
An example of the binary image: (**a**) Traffic direction estimation. (**b**) Binary image.

The side view geometric features of each scan can be obtained by projecting the PCD into a binary image along the direction perpendicular to the traffic direction of a bridge. First, the traffic direction of a bridge can be determined using the principal component analysis (PCA) algorithm. Considering the non-uniformity of scans, only the points that form the minimum convex hull of two-dimensional (2D) PCD on the *xOy* plane are utilized in the PCA algorithm. Second, the PCD is rotated so that its traffic direction aligns with the *x* axis, as shown in Figure 2a. Then, the 2D PCD can be obtained by projecting the PCD into the *xOz* plane. Next, the 2D PCD is voxelized using the grid sizes *δ_x_* and *δ_z_* (their calculation methods will be introduced later), and then it is converted to a binary image with each grid corresponding to a pixel. If the number of points within a grid is more than one, the grey value of the corresponding pixel is set to 1; Otherwise, it is set to 0. The binary image of the PCD in Figure 2a is shown in Figure 2b.

#### 3.1.2. Unified Scale of the Template and Binary Images

We employ an as-designed side view as the registration template. To ensure that the scale of the template is same as the binary images converted by PCD, the grid sizes *δ_x_* and *δ_z_* are determined using the following two formulas.

(1) $$\delta_{x}=\frac{L}{N_{x}}$$

(2) $$\delta_{z}=\frac{H}{N_{y}}$$

where *L* and *H* represent the length and height of the bridge, respectively; *N_x_* and *N_y_* are the width and height of the minimum bounding box of the non-blank region in the template.

#### 3.1.3. Image Matching

**Figure 3:**
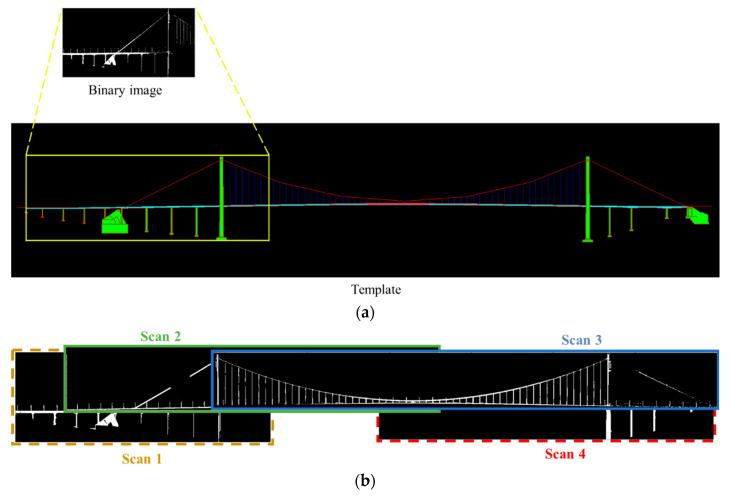
An example of image matching: (**a**) The relative positions of a scan with respect to the registration template. (**b**) The relative positions between different scans.

Image matching algorithms can be utilized to obtain the approximate positions of scans relative to the bridge. Based on the utilized primitives, image-matching algorithms can be classified as area-based matching (ABM) or feature-based matching (FBM) algorithms [24]. ABM is based on the idea that grey values of pixels of conjugate points have similar radiometric characteristics [25], while FBM is based on feature extraction, feature description, and correspondence feature matching. We opted for the ABM for image matching, which has high efficiency and reliability [26]. Given two images, ABM considers one as the reference image, the other one as the matching image. The matching image slides within a search window on the reference image, and a similarity measure is calculated at each position. The location of the matching image is assumed to be the position of the best agreement [27]. In our study, the registration template is set to the reference image and the search window is the entire image. Binary images are the matching images. An example of image matching is shown in Figure 3a. Considering that the image matching step can only determine the positions of PCDs but cannot adjust their orientations, the matching score between the horizontally mirrored image of the binary image and the registration template is also calculated. The case with the higher matching score is selected as the final result. Based on the matching relationship between each scan and the registration template, the relative positional relationships on the *xOz* plane between different scans can be obtained, as shown in Figure 3b. The alignment in the *y*-axis direction is determined based on the centres of the scans.

#### 3.1.4. Correction of False Matching

**Figure 4:**
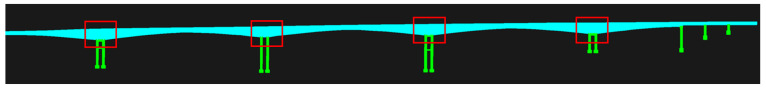
Repeated geometric patterns in a bridge.

In some cases, same geometric patterns may appear multiple times in a bridge, which may result in false matching. As shown in Figure 4, the geometric elements within the red boxes appear four times. When the coverage area of a scan is small, these repeated patterns may cause false matching.

To address this issue, a method for identifying and correcting false matching is proposed. A good scanning plan should provide a uniform coverage of a bridge while avoiding over-coverage in any particular area. When a scan is matched to a false location, the scan is considered “redundant” for the false location because it results in an over-coverage for the false location. Therefore, we can quantify the redundancy of a scan by comparing it with its adjacent scans. For the *i*th scan, its projected image is denoted as *I_i_*, and its areas intersecting with all the other scans are calculated based on the locations obtained in the template matching step. The *n* scans with the largest intersection areas are selected, their projected images denoted as *I*_1_, *I*_2_, …, *I_n_* are utilized to calculate the redundancy score *S_i_* for the *i*th scan using Formula (3).

(3) $$S_{i}=\frac{\left(I_{1}\cup I_{2}\cup \cdots \cup I_{n}\right)\circ I_{i}}{\sum_{a=1}^{A}\sum_{b=1}^{B}I_{i}\left(a,b\right)}$$

where *A* and *B* are the height and width of *I_i_*, respectively; (∘) denotes the Hadamard product. The scores for all the scans are then statistically analyzed to calculate the coefficient of variation. A higher coefficient of variation indicates a greater discreteness of scans, meaning false matching may be present. The top 20% scans with the highest scores are selected as potentially mismatched PCDs, awaiting further validation.

**Figure 5:**
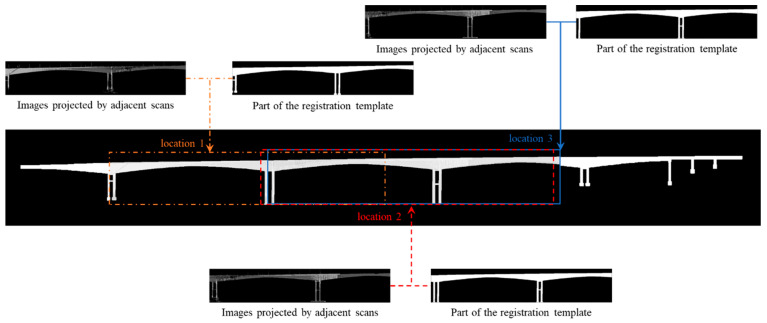
Candidate positions of a scan.

Considering that there may be differences between the as-designed side view and the actual state of the bridge, the similarity between a scan with its adjacent scans in the right location will be higher than the similarity between the scan with its adjacent scans in the false location. Therefore, further validation of whether a scan is mismatched can be determined based on its positional relationship with adjacent scans. For the *i*th scan which is labelled as potentially mismatched, three locations with the top three highest matching scores in the template matching step are considered as candidate positions. All the candidate positions are the pixels with the highest matching scores in a certain neighbourhood area. For each candidate position, the intersection areas with other scans that have not been labelled as mismatched are calculated. Three scans with the largest intersection areas are selected, and their projected images are added to the registration template with a proportion of 5% to modify the template, as shown in Figure 5. The matching score between the modified registration template and the *i*th scan is computed at each candidate position. The candidate position with the highest matching score is selected as the final position for the *i*th scan. 

### 3.2. Overlap-Based Scan-Block Construction

The main focus of our work lies in the efficiency of processing large bridges, such as suspension bridges, which may require dozens of TLS scans. The fully connected graph that arises from all these scans can be quite large, making the registration task computationally intensive. To address this challenge, a hierarchical registration strategy can be employed. This strategy can recursively subdivide and fuse the heavy registration task into smaller, more manageable subsets. By breaking down the registration process into hierarchical levels, the number of pairwise registration procedures can be significantly reduced [12]. In more detail, the hierarchical registration strategy involves partitioning all the scans into different scan-blocks and then locally aligns the scans in each scan-block, and finally performs the global block-to-block registration. The local registration accuracy of each scan-block plays a vital role in determining the overall registration accuracy of all the scans. To improve the registration accuracy, it is crucial to ensure significant overlaps within each block. Therefore, the block partitioning step plays a critical role in improving both registration precision and efficiency.

Partitioning scan-blocks must take into account both the scanning locations and overlaps between scans, and the overlaps of two scans can be qualified by the overlaps of their bounding boxes. The normalized cut (*Ncut*) algorithm [28] is utilized in our work for scan-block construction. First, a fully connected graph is created with each scan treated as a node. The edge weight *w_ij_* between the nodes *v_i_* and *v_j_* is defined as

(4) $$w_{ij}=\frac{IoU}{\Delta x+\Delta z}$$

where Δ*x* represents the ratio between the difference in *x*-coordinate values of the scanner positions and the width of the bridge, and Δ*z* the ratio between the interpolated *z*-coordinate values and the height of the bridge. *IoU* is defined as the intersection ratio of the bounding boxes of two PCDs, i.e.,

(5) $$IoU=\frac{B_{i}\cap B_{j}}{B_{i}\cup B_{j}}$$

*B_j_* and *B_i_* in Equation (5) represent the bounding boxes of two scans.

The objective of the *Ncut* algorithm is to minimize the cut between different blocks while maximizing the sum of the edge weights within each block. The objective function can be formulated as

(6) $$Ncut\left(A_{1},A_{2},\cdots ,A_{k}\right)=\sum_{i=1}^{k}\frac{cut\left(A_{i},{\bar{A}}_{i}\right)}{vol\left(A_{i}\right)}$$

where *A_i_* represents the *i*th scan-block, ${\bar{A}}_{i}$ the complement of *A_i_*, *k* the total number of blocks, and *cut*(*A_i_*, *A_j_*) the cut between *A_i_* and *j*th scan-block *A_j_*, i.e.,

(7) $$cut\left(A_{i},A_{j}\right)=\sum_{i\in A_{i},j\in A_{j}}w_{ij}$$

*vol*(*A_i_*) represents the sum of degrees of each node within *A_i_*, i.e.,

(8) $$vol\left(A_{i}\right)=\sum_{i\in A}d_{i}$$

**Figure 6:**
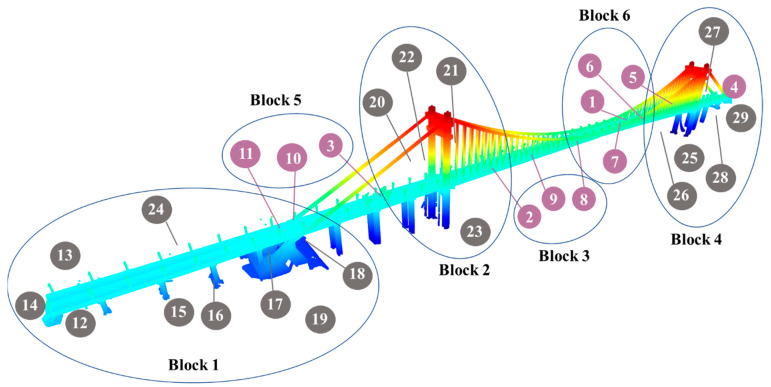
An example of the scan-block construction result.

Based on the normalized Laplacian matrix, the objective function can be simplified, and the Rayleigh–Ritz theorem can be employed for the solution. Finally, the partitioning results can be obtained using the *k*-means clustering algorithm [29]. Using the TLS scans of a suspension bridge as an example, the scan-block construction result is shown in Figure 6, where the numbers within the purple circles indicate the scanner positioned above the bridge deck, while the numbers within the black circles represent the scanner positioned below the bridge deck. It can be observed that our block construction method is capable of grouping scans with closer proximity and higher overlap into the same block.

### 3.3. Pairwise Coarse Registration by Optimization Algorithms

The pairwise coarse registration is commonly formulated as the maximum consensus set (MCS) problem [12]. Given two scans, *P* and *Q*, the point *p_i_* in *P* is paired with its nearest neighbour *q_j_* in *Q*, forming a point pair (*p_i_*, *q_j_*). The set of point pairs is represented as *H* = {(*p_i_*, *q_j_*)}_1_*^k^*, where *k* is the number of point pairs. If the distance between *p_i_* and *q_j_* is less than a threshold *δ*, the point pair is considered as a true correspondence. The MCS problem [30] aims to find the transformation matrix corresponding to the maximum number of true correspondences, i.e.,

(9) $$\underset{R,t,I\subseteq H}{\max}\left|I\right|,\;subject\;to\;\left\|p_{i}-\left(Rq_{j}+t\right)\right\|<\delta ,\;\forall \left(p_{i},q_{j}\right)\in I$$

The feature-based coarse registration method is the most commonly used coarse registration method [20]. It first extracts key points from two PCDs, and computes features of key points. Then a subset of key points is randomly selected from one PCD, and their corresponding points are searched in the other PCD using the feature similarity. After that, a transformation matrix can be obtained based on the matched point pairs, along with the number of true correspondences, which is called a consensus set. This process is usually iterated multiple times, and the transformation matrix associated with the highest number of true correspondences, which is called the maximum consensus set, will be output as the final transformations. However, existing feature extraction methods are susceptible to variations in point density and noise, and it makes the above methods less robust [20]. Considering that all the scans already have rough relative position relationships after the template matching step, performing pairwise coarse registration on the basis of this can significantly reduce the search space. We employ the particle swarm optimization (PSO) algorithm to search for the maximum consensus set.

The PSO algorithm is an iterative intelligent optimization algorithm that relies on collaboration and information sharing among particles to search for the optimal solution. During the searching process, each particle records its current position as well as its historical best solution, and the population also records its historical best solution. Based on the historical best solutions, the positions and velocities of the particles are updated, enabling the particle swarm to iteratively evolve and converge towards the global optimum. In this study, the particles denote the combinations of ***R*** and ***T***, and each particle is described by speed *v*_ij_ and position *x*_ij_, which are updated by Equations (10) and (11).

(10) $$v_{ij}(t+1)=wv_{ij}(t)+c_{1}\times r_{1}\times (p_{ij}-x_{ij}(t))+c_{2}\times r_{2}\times (p_{gj}-x_{ij}(t))$$

(11) $$x_{ij}(t+1)=x_{ij}(t)+v_{ij}(t+1)$$

where *i* is the particle number and *j* is a variable dimension; *v*_ij_(*t*) and *v*_ij_(*t* + 1) are the particle speeds at times *t* and *t* + 1, respectively; *x*_ij_(*t*) and *x*_ij_(*t* + 1) are the particle positions at times *t* and *t* + 1, respectively; *p*_ij_ is the historical optimal solution of the current particle; and *p*_gj_ is the historical optimal solution of the swarm; *c*_1_ and *c*_2_ are acceleration constants and both set to 2; *w* is the inertia weight and is set to 0.8; *r*_1_ and *r*_2_ are random numbers in the closed interval [0,1].

**Figure 7:**
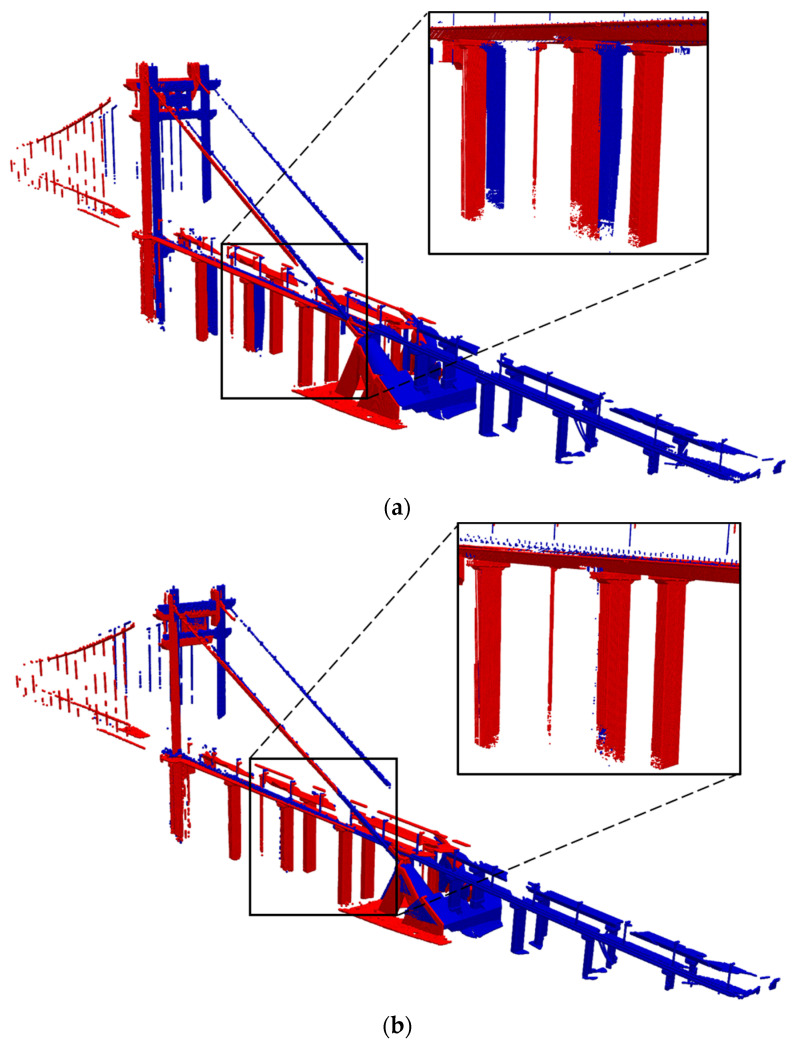
An example of coarse registration by the PSO algorithm: (**a**) Before PSO. (**b**) After PSO.

For the initial pose shown in Figure 7a, the PSO algorithm can obtain an accurate transformation, as shown in Figure 7b. Although the PSO algorithm can avoid becoming trapped in local optima, there may still be cases where two scans within the same scan-block cannot obtain the correct transformation matrix due to low overlap or slightly larger scanning distances. To identify false matches, Wu et al. [12] proposed a method based on the loop closure constraint and proved to be effective. This method is utilized in this study to reject false scan-to-scan matches obtained by the PSO algorithm.

### 3.4. Fine Registration and Pose Optimization

To obtain the optimal merging order of scans within each scan-block, the minimum spanning tree (MST) is utilized to extract a cycle-free and well-pairwise-registered graph [30]. The MST relies on edge weights to define the shortest path. In this study, the weight of each edge is defined as the number of true correspondences after the pairwise coarse registration, and the weights of the edges with false matches are set to 0. Along the edges of the MST, all the other nodes in a scan-block can be merged into the root node using the coarse registration matrix calculated by the PSO algorithm and the fine registration matrix computed by the ICP algorithm [31]. However, the errors will accumulate along the edges from the root to the leaf node since the MST is a cycle-free graph [13]. To address the issue of error accumulation, the Lu–Milios algorithm [32] is utilized to further optimize the pose.

Treating each scan-block as a new scan, the registration between scan-blocks can be accomplished using the same methods described in Section 1, Section 2, Section 3 and Section 4. This process is repeated until all scans are merged into a single scan. To avoid excessive point growth, PCD down-sampling is performed after the scan merging step.

## 4. Experiments and Analysis

### 4.1. Datasets Description and Evaluation Criteria

**Figure 8:**
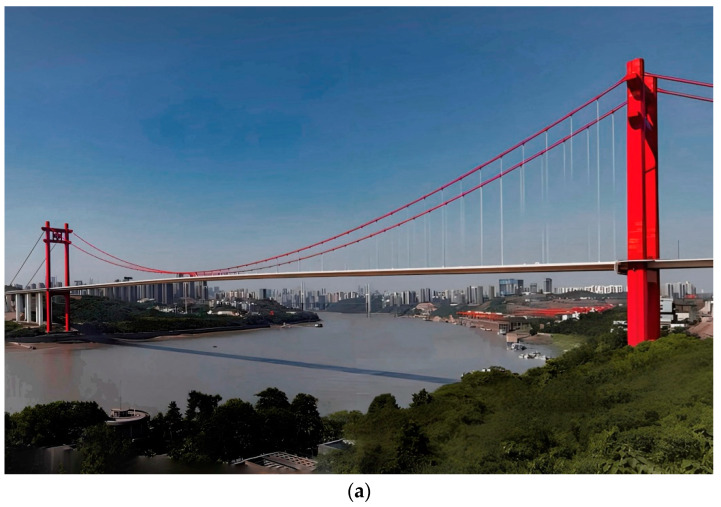

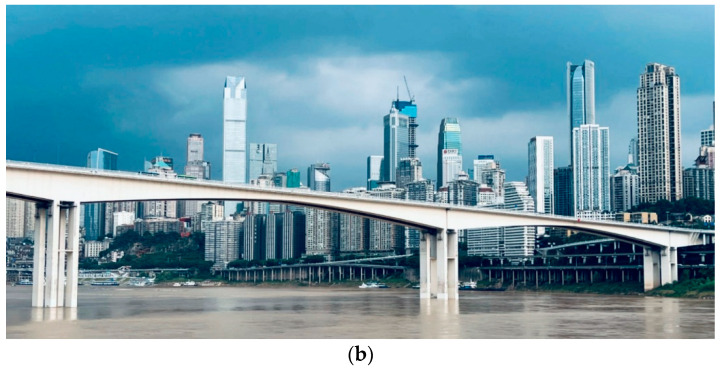
Two bridges in the bridge datasets: (**a**) Cuntan Yangtze River Bridge. (**b**) Huanghuayuan Jialing River Bridge.

We evaluated the performance of the proposed method using two bridge point cloud datasets, including a suspension bridge and a continuous rigid frame bridge. The suspension bridge named Cuntan Yangtze River Bridge is located in Chongqing with a total length of approximately 1.6 km and a width of about 38 m (Figure 8a). It was scanned using Leica P40 with a ranging error of 1.2 mm + 10 ppm and an angular accuracy of 8″ [33]. The continuous rigid frame bridge named Huanghuayuan Jialing River Bridge is located in Chongqing with a total length of approximately 1.2 km and a width of about 31 m. It was scanned using Faro S350 with a ranging error of 1 mm between 10 m to 25 m and an angular accuracy of 19″ [34]. More details about the two datasets are listed in Table 1.

For the Huanghuayuan Jialing River Bridge, only the area below the bridge deck was scanned using TLS, while the bridge deck area was scanned using a mobile scanning system. Prior to inputting the data into the algorithm, background points were roughly removed. In addition, the ground truth result was obtained through manual registration.

The performance of the method is evaluated by the axis-angle rotation error *e^r^* and translation error *e^t^* of all transformations among the scans [35], which are as follows:

(12) $$e^{r}=\arccos\left(\frac{tr\left(R^{g}{\left(R^{e}\right)}^{T}\right)-1}{2}\right)$$

(13) $$e^{t}=\left\|t^{e}-t^{g}\right\|$$

where *R^e^* and *t^e^* represent the estimated rotation matrix and translation vector, respectively, and *R^g^* and *t^g^* are the those of the ground truth. In addition, the successful registration rate (SRR) is also utilized and defined by

(14) $$\mathrm{SRR}=\frac{N_{s}}{N-1}$$

where *N* represents the total number of scans, and *N_s_* the number of successfully aligned scans. A scan is considered successfully aligned when its rotation error and translation error are both less than the specified thresholds *σ^r^* and *σ^t^*, respectively.

### 4.2. Results of Template-Guided Initial Pose Estimation

**Figure 9:**
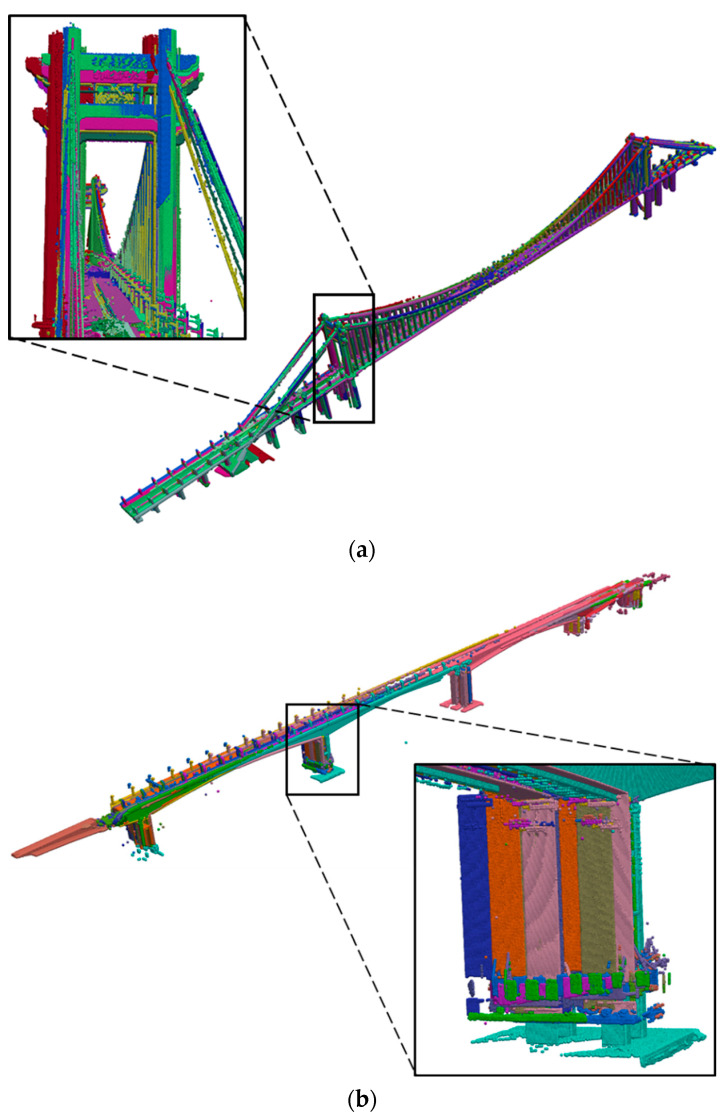
Initial pose estimation results of two bridges: (**a**) Cuntan Yangtze River Bridge. (**b**) Huanghuayuan Jialing River Bridge.

Based on the bridge datasets, we evaluated the accuracy and efficiency of the template-guided initial pose estimation method. The proposed method is implemented in Python through an Intel Core i7-7700K CPU (Intel, Santa Clara, CA, USA). The initial pose estimation results of the Cuntan Yangtze River Bridge and Huanghuayuan Jialing River Bridge are shown in Figure 9a and Figure 9b, respectively. It can be seen that this step can roughly align the scans and obtain the relative scanning positions.

The average rotation error, average translation error and running time for two bridges are listed in Table 2. For the Cuntan Yangtze River Bridge, the average rotation error is 19.1 mdeg, and the average translation error in the three coordinate axis directions are 0.66 m, 3.62 m, and 1.88 m, respectively. For the Huanghuayuan Jialing River Bridge, the average rotation error is 19.7 mdeg, and the average translation error in the three coordinate axis directions are 0.64 m, 5.16 m, and 2.35 m, respectively. Due to the alignment in the *y*-axis direction being solely based on the centres of the PCD, translation errors are more pronounced in the *y*-coordinate.

The running times of the two bridges are 6.08 min and 4.43 min, respectively. Compared with extensive pairwise matching, template-guided initial pose estimation has a higher efficiency, primarily due to two key reasons. Firstly, in our method, only *N* template matching is required to create a graph, where *N* represents the number of scans. In contrast, extensive pairwise matching necessitates $C_{N}^{2}=N\left(N-1\right)/2$ matches. This reduction in the number of matches significantly improves the efficiency of our approach. Secondly, in template-guided initial pose estimation, the problem scale in each individual match is determined by the number of points in the scan, denoted as *n*. The fundamental operation is mapping each point to a 2D grid. As a result, the time complexity for processing a single scan in the initial pose estimation step is O(*n*), and the time complexity for processing all scans is O(*Nn*). The quadratic time complexity ensures that our method has high efficiency and is adaptable to larger datasets. In summary, the data showed that our method can achieve a relatively high alignment accuracy, provide good initial poses for subsequent steps, and it helps to improve efficiency by avoiding extensive pairwise matching.

### 4.3. End-to-End Performance Evaluation

During the experiments, we set the average number of scans in a scan-block to 5, the down-sampling voxel size to 0.1 m, and *σ^r^* and *σ^t^* to 100 mdeg and 100 mm, respectively. The experimental setup utilized a hybrid programming approach, with the template-guided initial pose estimation step implemented in Python and the remaining parts in C++. Our method was tested on a laptop with 32 GB RAM and an Intel Core i7-7700K CPU.

**Figure 10:**
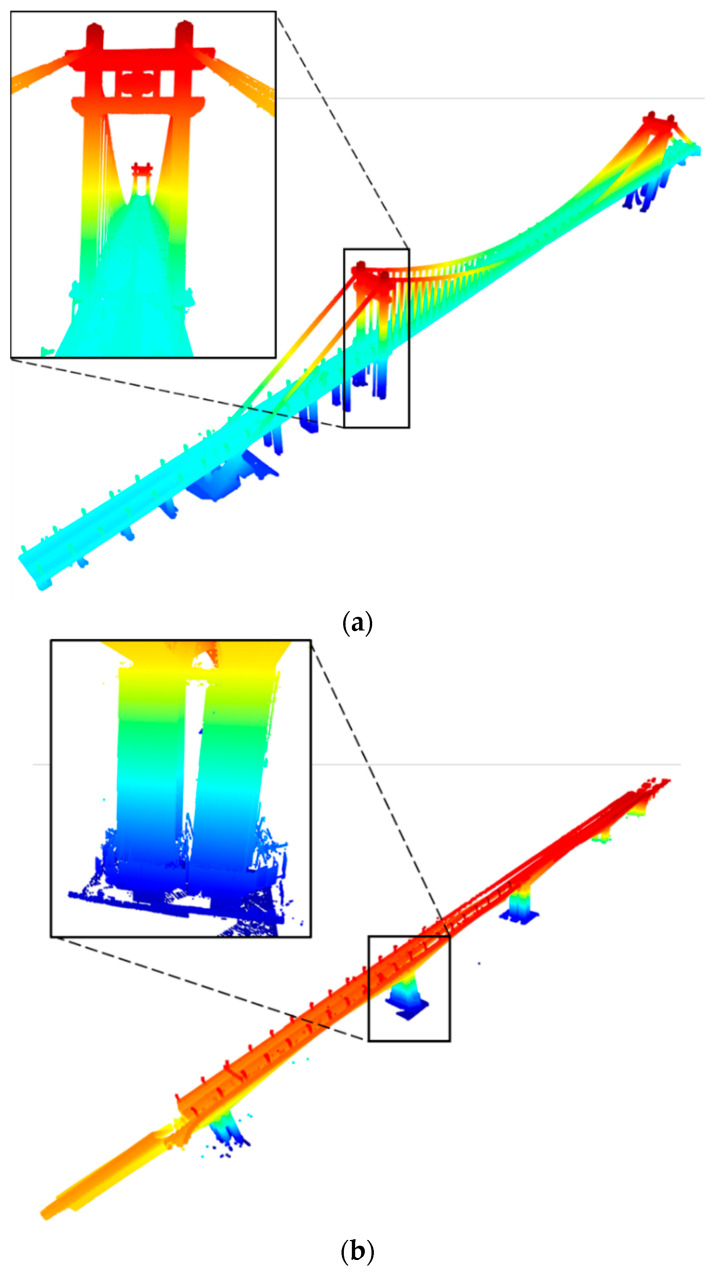
Registration results of the bridge dataset: (**a**) Cuntan Yangtze River Bridge. (**b**) Huanghuayuan Jialing River Bridge.

The registration results for the two bridges are plotted in Figure 10. The points are coloured according to the difference in height. It can be seen that the mismatching in Figure 9 has been removed. This demonstrates that our method can deal with PCD of large bridges, and obtain a good performance in accuracy.

The accuracy and efficiency of the proposed method was evaluated, and the rotation and translation errors with the root mean square error (RMSE), SRR, and running time are listed in Table 3. The average rotation errors of our method are 0.96, 0.74 (mdeg) and average translation errors are 28.04, 43.25 (mm), with the SSR of 100% and 100%. The results show that our method achieves relatively small rotation errors, but slightly larger translation errors. Compared with the Cuntan Yangtze River Bridge, the Huanghuayuan Jialing River Bridge has a relatively simple geometric shape and lacks complex features and structures, resulting a larger translation error. In conclusion, the registration results listed above prove that our method performs well in registering the TLS scans of varying bridges, and the accuracy can satisfy the requirements of component extraction and 3D reconstruction.

### 4.4. Discussion

Although our method has demonstrated good performance, it still has two limitations. Firstly, prior to registration, the manual removal of background points is required. This step can become cumbersome when dealing with a large number of scans. Therefore, future research will focus on developing automatic background-point removal techniques. Secondly, our method is currently only applicable to bridges, which limits its application. Future research will aim to expand this method to more diverse scenarios and environments.

## 5. Conclusions

In this paper, we present a template-guided hierarchical multi-view registration framework for aligning unordered bridge terrestrial laser scanning data without any markers. The proposed framework incorporates two distinct features: template-guided initial pose estimation and overlap-based scan-block generation. The former enables the rapid recovery of the scanning sequence for multiple unordered bridge scans, while the latter leverages the overlap between scans and a graph segmentation algorithm to partition the scans into different scan-blocks. Experimental results on two different bridge datasets demonstrate the accuracy and efficiency of the proposed method.

## Figures and Tables

**Table 1:** The detailed description of the two datasets.

Dataset	Scanners	Scans	Pts (Billion)
Cuntan Yangtze River Bridge	Leica P40 (Leica, Wetzlar, Germany)	29	1.52
Huanghuayuan Jialing River Bridge	Faro S350 (Faro, Lake Mary, FL, USA)	20	1.68

**Table 2:** Evaluation on template-guided initial pose estimation.

Dataset	Rotation Error (mdeg)	Translation Error (m)			Time (min)
		Δ*x*	Δ*y*	Δ*z*	
Cuntan Yangtze River Bridge	19.1	0.66	3.62	1.88	6.08
Huanghuayuan Jialing River Bridge	19.7	0.64	5.16	2.35	4.43

**Table 3:** Evaluation of the proposed method.

Dataset	Rotation Error (mdeg)		Translation Error (mm)		SSR (%)	Time (min)
	Average	RMSE	Average	RMSE		
Cuntan Yangtze River Bridge	0.96	0.67	28.04	14.13	100	50.6
Huanghuayuan Jialing River Bridge	0.74	0.66	43.25	28.48	100	40.5

## Data Availability

Data is unavailable due to privacy.
